# Unraveling the Bürgi-Dunitz Angle with Precision: The Power of a Two-Dimensional Energy Decomposition Analysis

**DOI:** 10.1021/acs.jctc.3c00907

**Published:** 2023-10-04

**Authors:** Israel Fernández, F. Matthias Bickelhaupt, Dennis Svatunek

**Affiliations:** † Departamento de Química Orgánica and Centro de Innovación en Química Avanzada (ORFEO−CINQA), Facultad de Ciencias Químicas, Universidad Complutense de Madrid, 28040-Madrid, Spain; ‡ Department of Chemistry and Pharmaceutical Sciences, AIMMS, Vrije Universiteit Amsterdam, De Boelelaan 1108, 1081 HZ Amsterdam, The Netherlands; § Institute for Molecules and Materials (IMM), Radboud University, Nijmegen 6500 GL, The Netherlands; ∥ Department of Chemical Sciences, University of Johannesburg, Johannesburg 2006, South Africa; ⊥ Institute of Applied Synthetic Chemistry, TU Wien, Getreidemarkt 9, 1060 Vienna, Austria

## Abstract

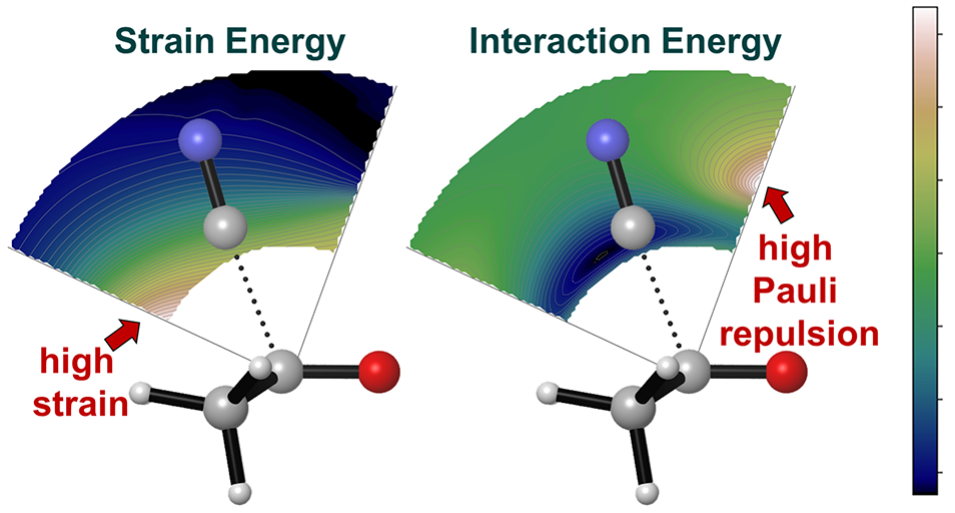

Understanding the geometrical preferences in chemical reactions is crucial for advancing the field of organic chemistry and improving synthetic strategies. One such preference, the Bürgi-Dunitz angle, is central to nucleophilic addition reactions involving carbonyl groups. This study successfully employs a novel two-dimensional Distortion-Interaction/Activation-Strain Model in combination with a two-dimensional Energy Decomposition Analysis to investigate the origins of the Bürgi-Dunitz angle in the addition reaction of CN^–^ to (CH_3_)_2_C=O. We constructed a 2D potential energy surface defined by the distance between the nucleophile and carbonylic carbon atom and by the attack angle, followed by an in-depth exploration of energy components, including strain and interaction energy. Our analysis reveals that the Bürgi-Dunitz angle emerges from a delicate balance between two key factors: strain energy and interaction energy. High strain energy, as a result of the carbonyl compound distorting to avoid Pauli repulsion, is encountered at high angles, thus setting the upper bound. On the other hand, interaction energy is shaped by a dominant Pauli repulsion when the angles are lower. This work emphasizes the value of the 2D Energy Decomposition Analysis as a refined tool, offering both quantitative and qualitative insights into chemical reactivity and selectivity.

## Introduction

Computational chemistry has been beneficial in providing insight into chemical reactions and offering valuable information for experimental work. Among the tools utilized in computational organic chemistry, the Distortion-Interaction/Activation-Strain Model (DI/ASM)^1^ coupled with Energy Decomposition Analysis (EDA)^2^ stands out. These methods are known for delivering both quantitative and qualitative insights into reactivity and selectivity. With a practical and adaptable approach, they have been employed in a variety of chemical contexts, ranging from refining textbook explanations^3−5^ to contributing to applied research in organic^6−9^ or main group^10^ chemistry and catalysis.^11−13^

The DI/ASM was pioneered by the groups of F. M. Bickelhaupt, who introduced the activation-strain model, and K. N. Houk, who contributed the distortion-interaction model.^1^ These two models within DI/ASM are equivalent and serve to dissect the change in energy Δ*E* of a chemical system composed of two distinct species, often termed fragments (e.g., reactants during a reaction), into two contributing factors:
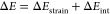


First, the strain energy, Δ*E*_strain_ (also known as distortion energy), quantifies the energy required to alter the fragments from their isolated relaxed geometry to the geometry adopted during interaction with the other species. Second, the interaction energy, Δ*E*_int_, accounts for the energy released upon bringing the two distorted fragments together.

Energy Decomposition Analysis can be used to further dissect the interaction energy into physically meaningful components. In this context, we employed the canonical EDA as implemented in the ADF software package. This method, which is based on the Ziegler-Rauk energy decomposition analysis,^14^ subdivides the interaction energy Δ*E*_int_ into three components:
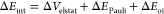


Δ*V*_elstat_ represents the electrostatic attraction of the charge distribution of one reactant with that of the other reactant. Δ*E*_Pauli_ characterizes the destabilization resulting from intermolecular filled-orbital/filled-orbital interactions and is responsible for steric repulsion. Δ*E*_oi_ accounts for the stabilization due to intermolecular filled-orbital/empty-orbital interactions and intrafragment polarization. For details, see ref (2).

Traditionally, these methods have been deployed to contrast the energetics between critical points, such as transition states, across different systems. However, it has been observed that this approach can sometimes yield data that is challenging to interpret or even lead to incorrect conclusions.^1,15,16^ One reason is that transition states can vary in terms of their progression, and a later transition state typically exhibits higher strain and interaction energies due to the closer proximity of the fragments. This proximity also significantly affects the EDA components, which generally intensify as the fragments draw nearer.

As a result, contemporary applications of DI/ASM and EDA often employ a “consistent geometry” approach, where specific geometric parameters are maintained consistently across systems, or they are conducted along a trajectory, such as a reaction coordinate.^1,15,16^ These refinements have markedly enhanced the clarity and reliability of insights garnered from such analyses.^7,17^

In a recent study, two of us (IF and FMB) explored the origin of the Bürgi-Dunitz (BD) angle in various nucleophilic addition reactions at carbonyl groups using quantitative Kohn–Sham molecular orbital (MO) theory and the Energy Decomposition Analysis.^18^ The Bürgi-Dunitz angle, typically around 107°, refers to the geometric angle at which a nucleophile approaches a carbonyl carbon during an addition reaction.^19−21^ This angle is considered stereochemically favorable for facilitating bond formation. Our research examined the energy differences between the optimal attack angle and the perpendicular (90°) attack using the interaction of cyanide with acetone as a model system. We compared an artificially created transition state with an attack angle constrained to 90° (**TS**_**90**_) to an unconstrained transition state (**TS**) at approximately 111°. Given the significant differences in bond length formation between these two transition states, the analysis was carried out along the two trajectories of 111° and 90°, comparing values at the same distance. Decreased Pauli repulsion, more favorable electrostatic interactions, and stronger orbital interactions were identified as the deciding factors. An EDA-NOCV (natural orbitals for chemical valence) analysis of the orbital interaction revealed a more favorable HOMO(nucleophile)-π*(C=O) LUMO interaction at 111°. Therefore, this study provided a comprehensive understanding and detailed analysis of why the 111° angle is preferred over 90° for this system.

Building on this foundation, we now propose extending this analysis to a 2D potential energy surface to gain a complete picture of the origin of the Bürgi-Dunitz angle. We are interested in not only what pushes the angle from 90° to higher values but also what limits the angle to even higher values. We introduce an enhanced method of employing DI/ASM and EDA—by applying them across multidimensional surfaces, with our focus on the 2D potential energy surface. Theoretically, this approach can be adapted to higher dimensions, providing a broader perspective of complex chemical interactions. With regard to the Bürgi-Dunitz angle, this methodology shines a spotlight on the nuanced factors that shape the potential energy surface. A clear advantage of this technique is its simplification of analysis. The intuitive graphical representation bypasses the need to trace numerous lines on a graph, which is a common challenge when analysis is restricted to a single reaction coordinate. Moreover, this approach allows the extraction of various 1D slices from the 2D surface, paving the way for a detailed quantitative inspection of key influences.

We demonstrate that an Energy Decomposition Analysis on a 2D plane offers a comprehensive, quantitative, and qualitative understanding of a range of potential attack trajectories. This approach serves as an effective tool for enhancing our understanding of intricate chemical interactions.

## Results and Discussion

**Figure 1:**
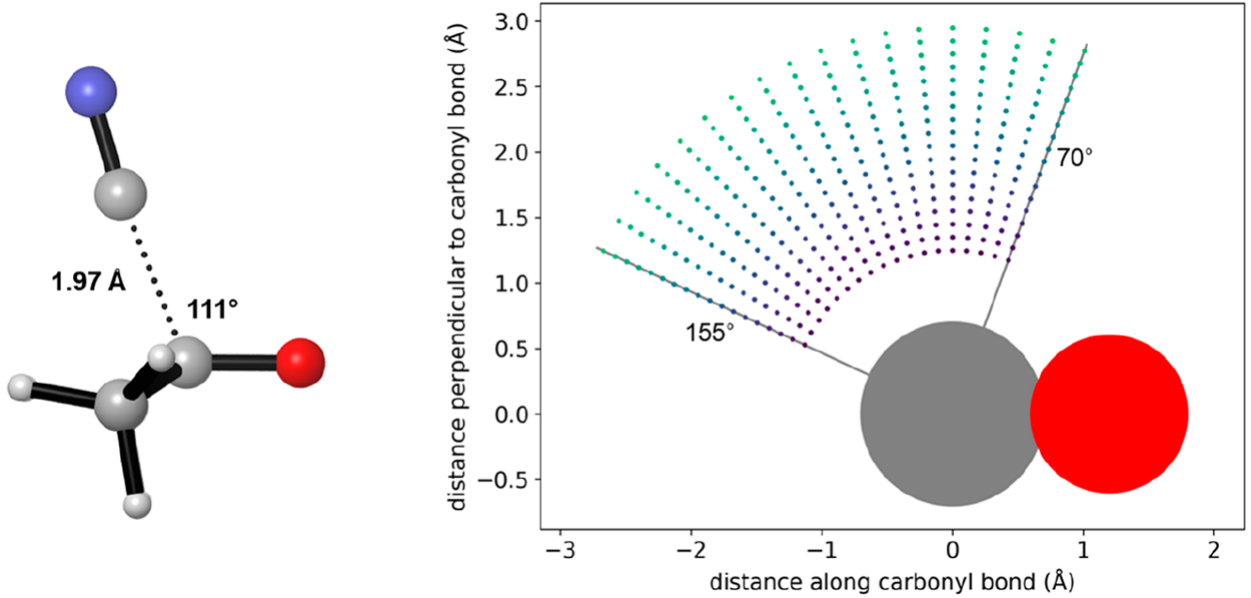
Transition state of the investigated reaction and schematic representation of the 2D energy surface grid composed of 324 structures, parametrized by the C–C distance (between cyanide and the carbonyl carbon) and the C–C–O angle (between the cyanide carbon, carbonyl carbon, and oxygen) for the cyanide/acetone model system. The carbonyl bond is aligned along the *x*-axis with carbon (gray sphere) at the origin and oxygen (red sphere) at 1.2 Å.

In our study, we employ the cyanide/acetone model system as a representative framework for the analysis. To investigate the potential energy surface associated with the attack of cyanide on acetone, we devised a grid encompassing 324 structures. This grid was laid out on a 2D surface, characterized by two principal geometric descriptors: the C–C distance spanning between the carbon of the cyanide and the carbonyl carbon in acetone (ranging from 1.25 to 2.95 Å) and the C–C–O angle formed by the alignment of the carbon in CN, the carbonyl carbon, and the oxygen in acetone (varying from 70° to 155°, Figure 1).

This comprehensive grid allowed us to meticulously examine the energy landscape as the geometrical parameters evolved, offering valuable insights into the interplay between distance and angular configurations during nucleophilic attack.

**Figure 2:**
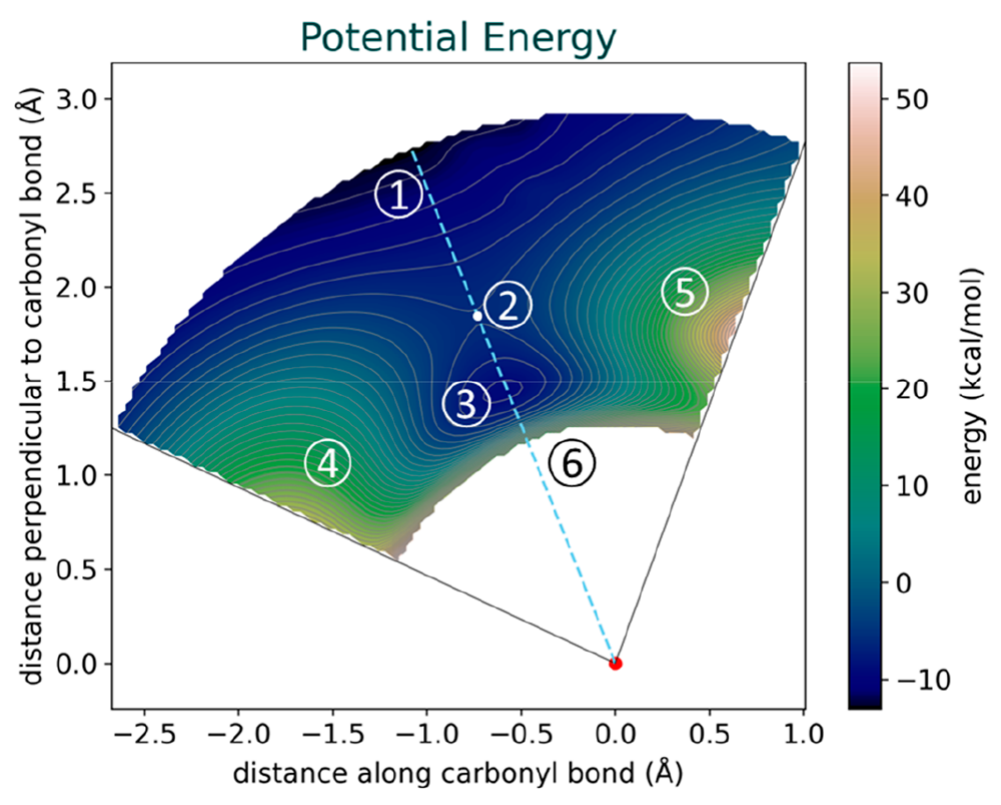
Potential energy surface (PES) for the cyanide/acetone interaction. The PES is divided into six distinct regions, with regions labeled 1–6 for reactant complex, transition state (saddle point marked with a white dot), product, high energy flanking regions, and atomic repulsion region, respectively. The light blue line shows the optimal attack angle of 111°.

The Potential Energy Surface (PES) spanning the aforementioned region is illustrated in Figure 2. Upon analysis, the PES can be divided into six discrete regions. Region 1 encompasses the reactant complex area. Region 2 denotes the transition state, with the saddle point distinctly marked by a white dot. Region 3 represents the product zone. Regions 4 and 5 are peripheral zones with elevated energy levels. These “flanking” zones are instrumental in shaping the product valley and also in establishing the energy barrier or saddle point that separates the reactants from the product. Lastly, Region 6 signifies the high-energy sector corresponding to short C–C distances and is predominantly influenced by direct interatomic repulsion.

**Figure 3:**
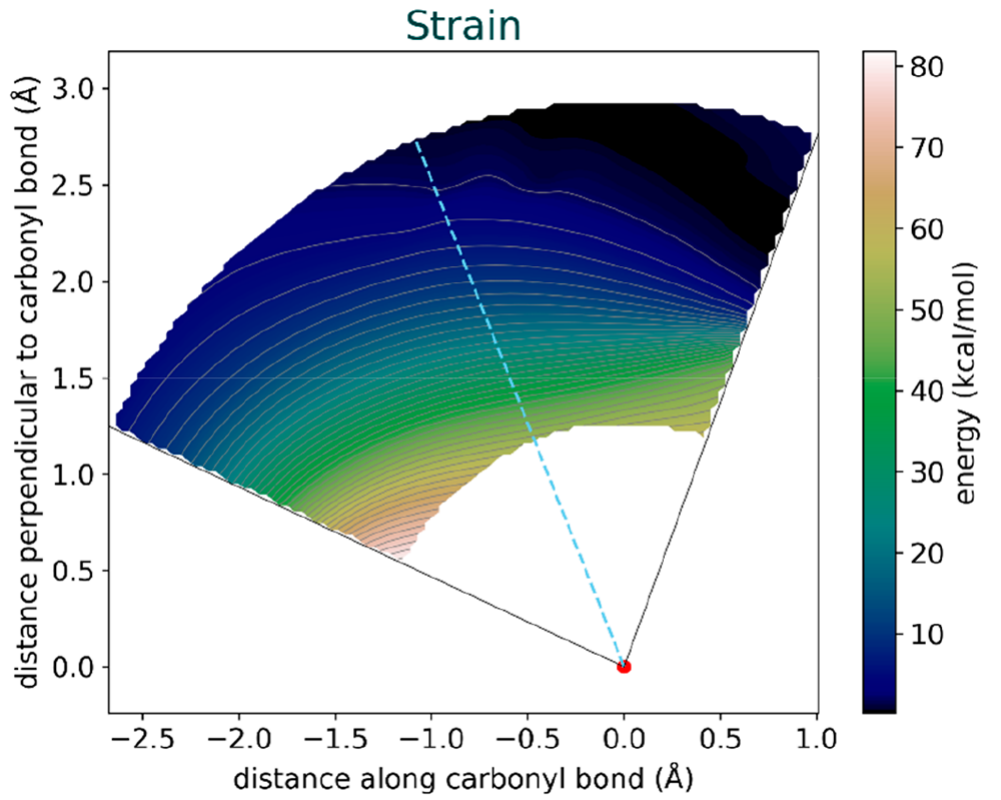
Strain energy surface for the cyanide/acetone interaction depicting the dependency of strain energy on the C–C distance and the C–C–O angle. The figure shows how the strain energy increases with decreasing intramolecular distance and also reveals angle-dependent components.

Subsequently, we carried out the Distortion-Interaction/Activation-Strain Analysis on this PES. This analysis yielded four additional energy surfaces, namely, the surfaces corresponding to the strain of each reactant, the cumulative strain energy, and the interaction energy. It is worth noting that the strain energy for the cyanide anion remains marginal, below 1 kcal/mol, throughout the evaluated surface. As a consequence, total strain energy is almost completely governed by the strain of acetone and will be investigated instead of analyzing strain for each reactant separately (Figure 3).

As expected, the strain energy increases significantly at shorter intramolecular distances. Additionally, we observed an angle-dependent characteristic; strain energy tends to be higher at larger angles for equivalent distances. This observation can be rationalized in terms of steric hindrances; at large angles, unfavorable Pauli repulsion arises between the incoming nucleophile and the methyl groups of acetone, leading to unfavorable interactions. However, the nucleophile can moderate some of this repulsion by adjusting its own distortion to minimize the total energy. The flexibility of the carbonyl compound thus becomes crucial, enabling the system to adapt to this Pauli repulsion, and hence, the resulting high-strain energy region emerges as a product of this Pauli-induced distortion. As such, the distinctive shape of the energy landscape is derived from the interplay between the adaptability of the carbonyl compound and Pauli repulsion.

**Figure 4:**
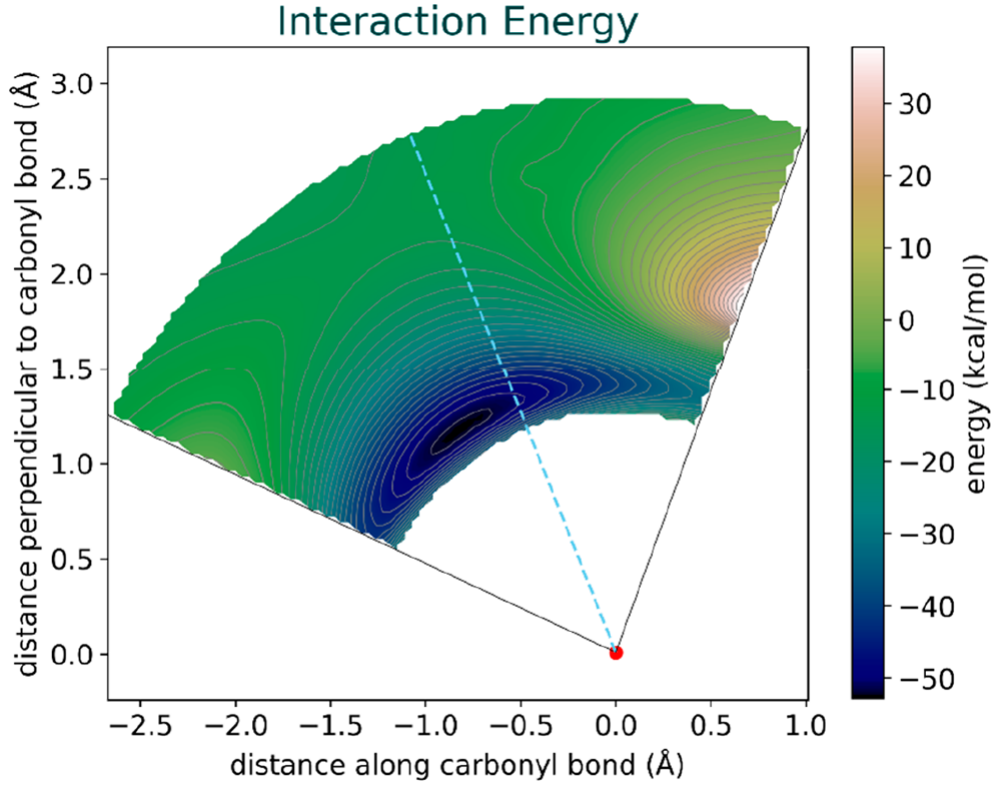
Interaction energy surface obtained from the Distortion-Interaction/Activation-Strain Analysis. The figure displays a minimum in energy at an angle of 125°, illustrating the influence of distortion energy on the angle of attack.

Figure 4 shows the energy surface for the interaction energy. Interestingly, there is an energy minimum at an angle of 125°, which is quite different from the angles at the product and transition state in the full PES. This suggests that the attack angle does not adopt higher bond angles mainly because of strain, as it is the main factor contributing to the high energy in Region 4 in Figure 2, while interaction energy alone would favor a larger angle than observed.

**Figure 5:**
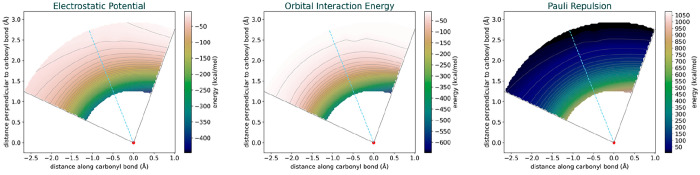
Energy Decomposition Analysis energy surfaces depicting electrostatic potential, orbital interactions, and Pauli repulsion. The plots illustrate the interplay between these energy components as a function of the C–C distance and C/C/O angle.

Additionally, there is a high-energy region in the interaction energy at lower angles and distances of about 2 Å. This region roughly corresponds to high energy Region 5 in the complete PES (Figure 2) and is responsible for diverting the 90° attack trajectory to higher angles. To delve into the intricacies of the interaction energy surface, it is imperative to conduct an Energy Decomposition Analysis that leaves us with three additional energy surfaces: electrostatic potential, orbital interactions, and Pauli repulsion (Figure 5).

All three EDA energy components exhibit a strong dependence on interfragment distance. The electrostatic potential and orbital interactions become increasingly favorable as the distance decreases, while the Pauli repulsion correspondingly becomes more repulsive. At first glance, the energy components appear to exhibit a minimal angular dependence. However, upon closer inspection, it is evident that they deviate from a perfectly circular pattern, particularly at smaller angles. This deviation suggests that there is a subtle angular dependence in the energy components in addition to the pronounced radial dependence.

**Figure 6:**
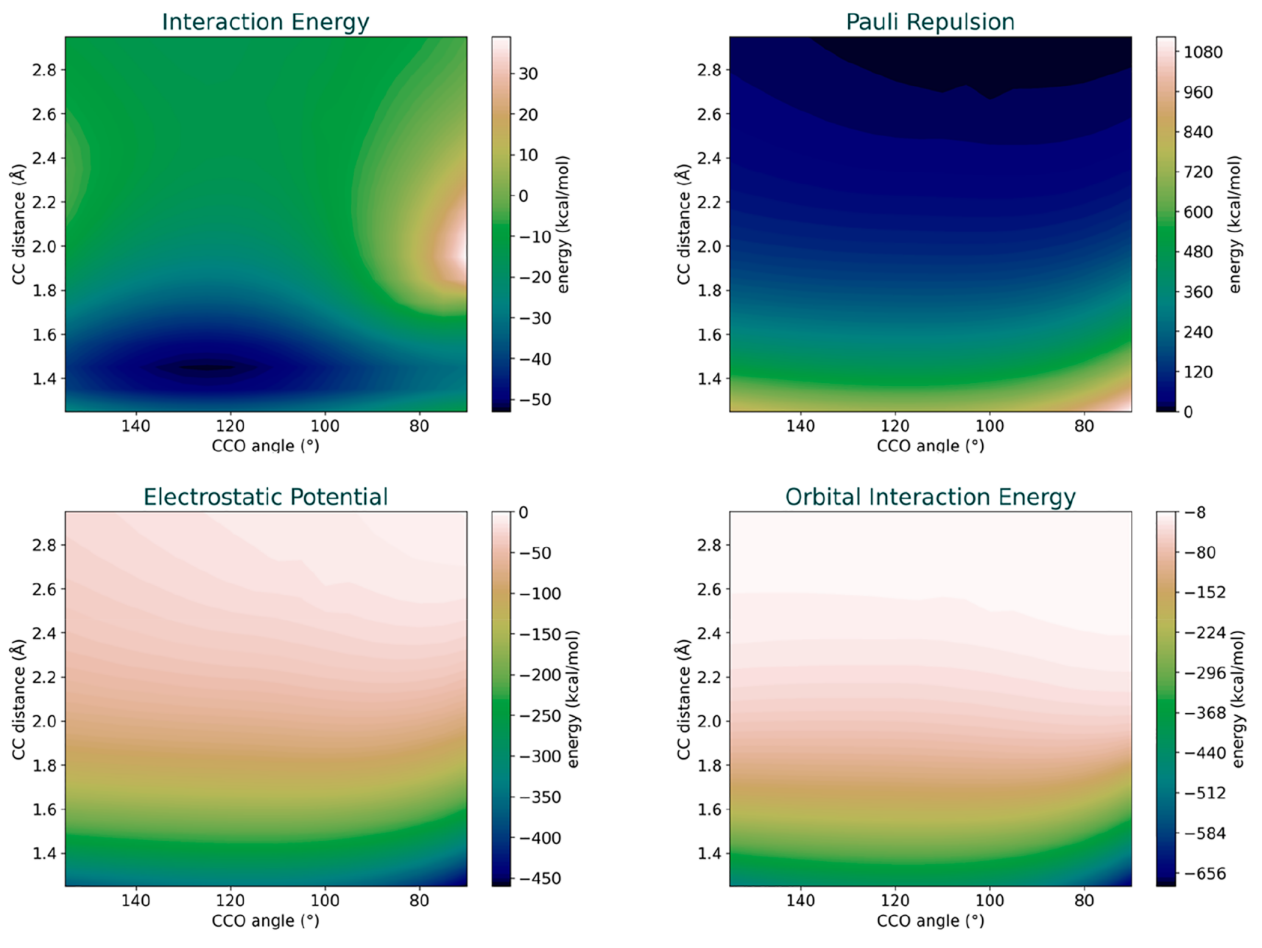
Radial distance vs angle plot for interaction energy and the EDA energy components, demonstrating the angular dependence of electrostatic potential, orbital interactions, and Pauli repulsion.

One of the challenges here is the choice of a coordinate system. Employing a plot with radial distance versus angle offers better clarity regarding the angular dependence (Figure 6).

This plot illustrates that the angular dependence is considerably prominent at smaller angles. The greater interaction at lower angles and longer distances signifies that the electrostatic potential, orbital interactions, and Pauli repulsions have a relatively stronger effect at these positions. This implies that as the interacting fragments are farther apart, they experience stronger interactions at lower angles compared to higher angles. All three components, electrostatic potential, Pauli repulsion, and orbital interactions, showcase this behavior.

However, discerning how the interplay of these components shapes the interaction energy surface is still challenging, particularly since the change in energies with the radial distance is much higher than the angular differences. This underscores the limitations of 2D EDA for visual analysis. Nonetheless, with the energy surfaces at hand, it is feasible to create cross sections of these surfaces in various orientations for quantitative investigations.

**Figure 7:**
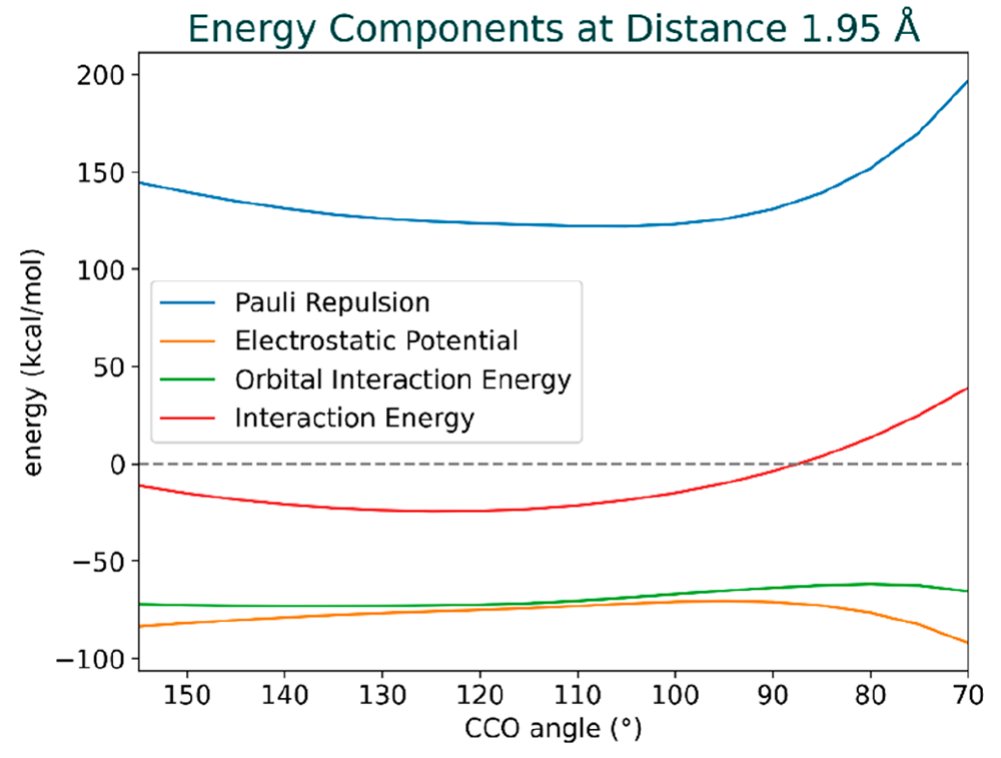
Cross-sectional data of interaction energy, electrostatic potential, orbital interaction, and Pauli repulsion surfaces at a fixed distance of 1.95 Å across angles ranging from 70 to 155 degrees. The figure highlights the dominance of the Pauli repulsion in shaping the overall interaction energy.

We now turn our focus to discern what gives rise to the high-energy region in the interaction PES at around a 2 Å distance and lower angles (70–90°), which consequently nudges the attack trajectory toward higher angles (Figure 6a). We dissect the interaction energy, electrostatic potential, orbital interaction, and Pauli repulsion surfaces along a fixed distance of 1.95 Å for angles ranging from 70 to 155 degrees. We opted for 1.95 Å since it is marginally shorter than the TS distance and is in proximity to where the interaction energy reaches its maximum (Figure 7).

Pauli repulsion appears relatively flat in the range between 130° and 100° but exhibits a sharp uptick between 100° and 70°. As for the electrostatic potential, we observe a slight, nearly linear decrease in stabilization from 155° to 90°, transitioning to a stronger stabilization trend as the angle approaches 70°. However, this increased stabilization is insufficient to counterbalance the growth in the Pauli repulsion.

For the orbital interactions, a modest decrease in the strength of attraction occurs as the angle draws near 80°. As the angle diminishes further beyond 80° toward 70°, the strength of the orbital interaction reestablishes, becoming more attractive. It is important to note that these variations in the orbital interaction strength are relatively minor when compared to the changes in Pauli repulsion.

By analyzing the data, we discern three segments in the Pauli repulsion curve that stand out. Below 90° and above approximately 125°, it is the Pauli repulsion that exerts control over the interaction energy. However, within the middle segment from 90° to 125°, the interaction energy seems more governed by the curves of the electrostatic potential and orbital interaction, which gradually become less favorable upon transitioning from 125° to 90°.

**Figure 8:**
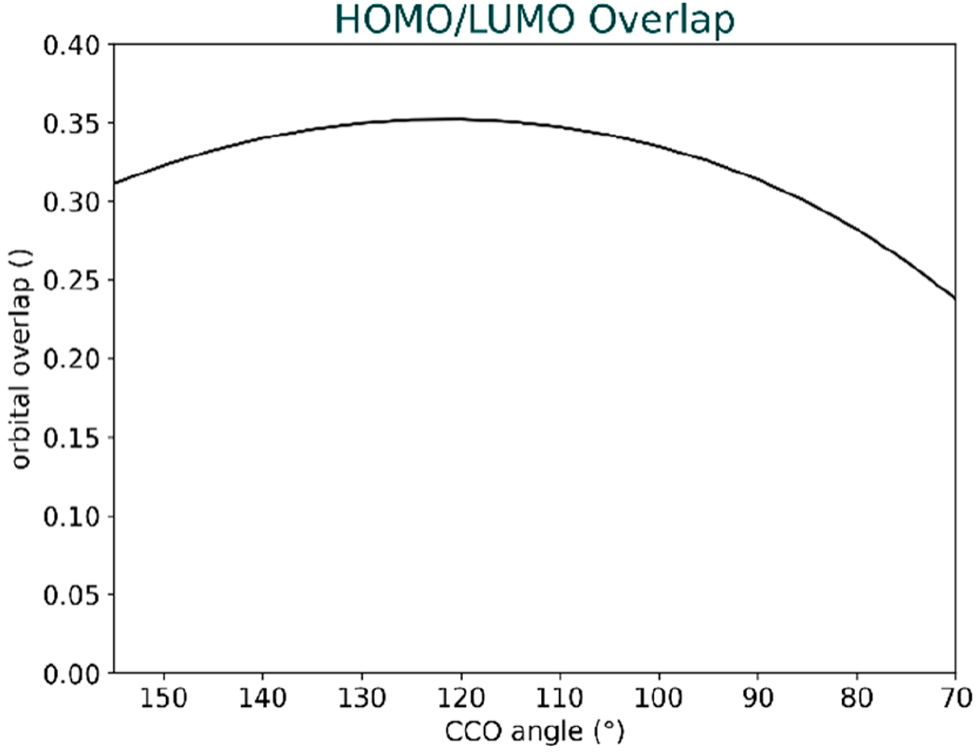
HOMO_cyanide_/LUMO_acetone_ orbital overlap at a fixed distance of 1.95 Å across angles ranging from 70 to 155 degrees.

To comprehend the nuanced behavior of the orbital interaction curve, we analyzed the overlap between the HOMO of nitrile and the LUMO of acetone (Figure 8). The maximum overlap occurs at around 120° with a slight decrease toward higher angles and a more pronounced decrease toward lower angles. This aligns with the analysis in the previous paper^13^ and offers further understanding of the preference for the Bürgi-Dunitz angle over a 90° approach.

**Figure 9:**
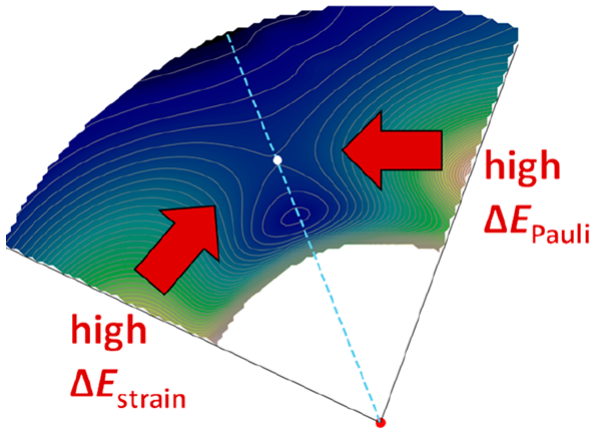
Illustration of the main factors locking the attack angle to 111° in the cyanide/acetone system.

Our findings reveal that the Bürgi-Dunitz angle is a result of a balance between the strain energy and the interaction energy. Notably, we observed that the strain energy limits the Bürgi-Dunitz angle from reaching higher values, which would have otherwise been favored by the interaction energy (Figure 9). This higher strain at a larger angle is the manifestation of steric Pauli repulsion between the nucleophile and the methyl substituents that is absorbed into a geometrical deformation of the acetone reactant.

The angular dependence of the interaction energy was further analyzed, and it was found that the Pauli repulsion dominates in shaping the overall interaction energy at the relevant distances. Specifically, an increase in Pauli repulsion at lower angles and interfragment distances around 2 Å forces the attack trajectory to higher angles. Note that, at these lower angles, Pauli repulsion is not converted into acetone deformation because, besides carbon, the carbonyl oxygen has no substituents to be involved in steric repulsion that could bend away from the nucleophile.

## Conclusion

We have explored the origin of the Bürgi-Dunitz angle through an insightful and comprehensive two-dimensional Energy Decomposition Analysis using the cyanide/acetone model system. The potential energy surface and the accompanying energy components, such as strain energy and interaction energy, were visualized across a wide range of possible attack trajectories. The graphical analysis provides an intuitive understanding of the various factors that influence the geometry of nucleophilic attack on carbonyl groups.

A remarkable aspect of this study is the successful application, for the first time, of the 2D distortion-interaction-activation-strain model and EDA, which proved to be invaluable in yielding both quantitative and qualitative insights into reactivity and selectivity. This two-dimensional approach extends the depth of analysis beyond traditional methods, allowing for more nuanced explorations of energy surfaces and offering a clearer understanding of the underlying energetic contributions. As demonstrated through analysis of the Bürgi-Dunitz angle, reactivity arises from a complex interplay of competing factors that achieve a balanced state. A multidimensional analysis is essential for accurately capturing the influences of different factors on a hyperdimensional energy surface.

We anticipate that this two-dimensional methodology will be especially useful in scenarios where it is difficult to define a common point for comparative analysis such as cycloaddition reactions with varying asynchronicities. It enables an unbiased examination of the potential energy surface, circumventing the bias that might be introduced by selecting specific reaction coordinates.

## Computational Methods

Geometry optimizations for the 2D potential energy surface scan were conducted using the Gaussian 16 software package,^22^ employing the M06-2X functional^23^ with the 6-311+G(d,p) basis set.^24^ For these calculations, relaxed scans were utilized, treating the C–C distance (between the cyanide and carbonyl carbons) and the C–C–O angle (between the cyanide carbon, carbonyl carbon, and oxygen) as constrained parameters. Following the geometry optimization, the Distortion-Interaction/Activation-Strain Model and Energy Decomposition Analysis were implemented using single-point calculations in the Amsterdam Density Functional (ADF) software.^25−27^ These calculations utilized the M06-2X functional with the TZ2P basis set,^28,29^ without frozen core approximation. The numerical quality was set to “very good”, and the scalar relativistic effects were accounted for using the Zeroth-Order Regular Approximation (ZORA).^30^

All scripts employed for the analysis are publicly accessible on GitHub at https://github.com/dsvatunek/2D_EDA/releases/tag/v1.0.0 and will be incorporated in the forthcoming release of our autoDIAS software package.^31^ All used geometries and obtained energies are provided as Supporting Information.

The computations were executed on a Windows workstation equipped with a 32-core AMD Ryzen ThreadripperTM 3970X CPU, with the entire computational process taking under 16 h. Notably, the 2D EDA proved to be computationally efficient, underscoring its practicality for similar investigations, even without the need for high-performance computing resources.

## Supporting Information Available

jmol file (XYZ)
Orbital overlaps (XLSX)
jmol file (XYZ)
jmol file (XYZ)
EDA energies (XLSX)

## Supplementary Material

ct3c00907_si_001.xyz

ct3c00907_si_002.xlsx

ct3c00907_si_003.xyz

ct3c00907_si_004.xyz
